# Hospitals and Flannelette

**Published:** 1911-05-27

**Authors:** A. F. Edwards

**Affiliations:** West Didsbury, Manchester.


*To the Editor of* The Hospital.

### HOSPITALS AND FLANNELETTE.

*To the Editor of* The Hospital.

Sir,—Your paragraph on "Hospitals and Flannelette" is a timely one, and with all you say I am in full agreement, save one statement which is a little vague. You say that in hospitals where flannelette is used "care is usually taken that the cloth is fire-proofed by one or other of the methods which are generally considered reliable." May I point out that the Report of the Commission which recently dealt with the flannelette question distinctly says that only one method of fire-proofing flannelette meets with their approval, namely, the Non-flam process, which renders the material *permanently* fire-proof.

This had been put to the most exhaustive tests by the Government Analyist, acting on behalf of the Commission; by the British Fire Prevention Committee; and by a group of scientists. Evidence to this effect was also put before the Commission by several coroners and others who had made similar tests of the material for themselves.

As to the *temporary* methods of fire-proofing, such as the use of alum, etc., these were all condemned by the committee in their report, and by every witness whose opinion upon the matter was asked, on grounds of cost, of unreliability, and of a tendency of such salts to render the flannelette hygroscopic, and thus create a new danger to the wearer. I have tried the one given only last week, in a "Domestic Economy" column, using the amount stated, and also twice that strength. On submitting these to three witnesses independently, they could tell no difference between treated and untreated—in one case saying the specimen with twice the quantity was, if anything, slightly more inflammable than that not treated at all! I enclose specimens for your examination.—Yours faithfully, A. F. Edwards.

West Didsbury, Manchester.

[The specimens enclosed were certainly not non-inflammable, although they had been treated with alum, and were in striking contrast to the "Non-flam" samples sent.—Ed. The Hospital.]

